# Socio-demographic and cross-country differences in attention to sustainable certifications and changes in food consumption

**DOI:** 10.1038/s41538-024-00274-x

**Published:** 2024-06-01

**Authors:** Jatziri Mota-Gutierrez, Antonina Sparacino, Valentina Maria Merlino, Simone Blanc, Filippo Brun, Fabrizio Massimelli, Emanuela Vassallo, Danielle Borra, Stefano Massaglia

**Affiliations:** 1 Department of Veterinary Sciences, University of Turin, Largo Paolo Braccini 2, 10095 Grugliasco, Turin Italy; 2 Department of Agricultural, Forest, and Food Sciences, University of Turin, Largo Paolo Braccini 2, 10095 Grugliasco, Turin Italy; 3 Independent Researchers, Via Vivaro 21/B, 12051 Alba, Italy

**Keywords:** Environmental impact, Environmental economics, Sustainability, Climate-change impacts, Economics

## Abstract

Food labeling can influence, sometimes facilitate, changes in consumer diets to support environmental sustainability and in response to climate change. However, a significant impediment to this dietary shift may arise from the consumers’ tendency to underestimate the environmental impact of their food choices and from their limited knowledge about sustainable certifications. These aspects are influenced by the characteristics and geographical affiliations of individuals. In such a context, the aim of this research has been to identify the main factors that drive the food purchasing frequency and the changes in food consumption associated with consumers’ concerns about climate change and interest in sustainable food certifications by comparing different food products and countries (Italy, France, Germany, Denmark, the USA, and China). A cross-country survey was conducted on 6500 consumers of various food products. The obtained mean scores were then compared, using generalized linear mixed-effect models, to evaluate the associations between the consumers’ food purchasing frequency, the importance of sustainable certifications, and changes in food consumption due to climate change concerns. Much of the variation in food consumption, purchasing behaviors, and interest in sustainable certifications was found to depend on such factors as age, gender, and country of origin. Indeed, Chinese consumers exhibited a heightened interest in sustainable food certifications, yet their consumption scores for all food products overall were higher. Conversely, adult and elderly Danish consumers demonstrated a decrease in the consumption of cheeses, meat, fruits, and vegetables, and their interest scores in all sustainable food certifications were lower. Despite the challenges posed by various consumers’ interests and minimal changes in food consumption patterns, our findings suggest that sustainable certifications present a promising avenue for straightforward interventions to promote the adoption of sustainable diets and to address climate change.

## Introduction

Sustainable consumption involves adopting food behaviors that result in minimal negative environmental impacts an potentially in turn livelihoods of future generations^1,2^. Some of the drivers that influence consumers’ attitudes and sustainable food choices include social media and certification labels^3^. According to^4^, labels and certifications play important roles in influencing sustainable behavior, increasing knowledge, creating favorable opinions about sustainable products, and promoting dietary changes to reduce climate change^5^. “Sustainability” and “sustainability certifications” can both be considered as credence attributes that are currently influencing consumers’ sustainable food choices^6–8^. The aim of sustainability labels is to distinguish pro-environmental from pro-social brands^9^ and to attract consumers without arousing skepticism^10^. However, consumers mainly associate the concept of “sustainability” with such environmental issues as greenhouse gas emissions or water use, and they tend to associate ethical and social sustainable consumption and food processing less with it.

Sustainable food choices are influenced by a complex interaction between consumers’ environmental concerns and their level of awareness and knowledge of the sustainability certifications of different types of products and of different food origins. According to ref. ^11^, consumers in that period showed greater interest in sustainability certifications of meat than of other products, and 70% of the participants in their investigation believed that sustainable production certifications were important^11^. Moreover, vegetables seem to arouse environmental concerns, with ref. ^3^ indicating that 60% of their participants considered the environmental impact of vegetables to be a key purchasing factor^3^. The situation is more nuanced in the case of cheeses, where consumers often associate sustainability certifications with organic cheeses, and the environmental concern about this product is not as pronounced as it is for meat or vegetables^12^. Moreover, a recent study has shown that age, education, gender, social background, geographical location, and political factors are all determinants of consumers’ attitudes and sensitivity toward the impact of the climate on food^13^.

Geographical affiliation, which determines the different socio-political, cultural, and situational characteristics of individuals, has a great impact on consumers’ involvement in climate change and food production^14–16^. Research on the impact of individuals’ attention to climate change on food consumption patterns in different countries provides valuable insights into the relationship between the food choices and active pro-environmental involvement of comsumers^17,18^. Changes in the nutritional aspects of consumers’ diets, caused by the impact of climate change, and interest in sustainable certifications have already been studied elsewhere^17,19–21^. Existing research shows that consumers around the world are increasing their consumption of animal-based products, particularly meat, processed food, and dairy products, thereby contributing to increasing greenhouse gas emissions, deforestation, and land degradation^22–24^. Studies in France show that the average French diet has a relatively high carbon footprint, due to the consumption of animal-based products, particularly beef and dairy products. However, there is a growing trend among French consumers to adopt flexitarian and plant-based diets, which may help reduce the overall environmental impact of food consumption in this country^25^. Research suggests that Danish consumers prioritize environmental and ethical considerations when choosing products^26,27^. In this country, consumers have adopted sustainable eating habits, preferring more organic and local food. Instead, China’s economic and population growth has led to significant changes in the population’s food consumption habits, which have resulted in an increased demand for meat and animal products, even from other continents^28^.

Studies indicate that the sensitivity of consumers to environmental sustainability during food purchases differs across regions and demographic groups^29,30^. After the COVID-19 pandemic, Chinese women started to purchase more environmentally friendly food than men^31^; however, it was observed that there were differences in attention to sustainable food certification according to age. In particular, the younger population showed a greater awareness of environmental issues and made more conscious sustainable choices on the basis of certifications^32^. Thus, it is crucial to understand how socio-demographic factors influence consumers’ perceptions of sustainable food certifications and food purchases in different geographical regions.

At the same time, the levels of awareness, interest, and trust in sustainable certifications play significant roles in influencing the purchasing decisions of consumers^33–35^. Italian consumers have shown an increasing interest in environmental sustainability in the food sector^36,37^. According to a recent research, 75% of Italian consumers are willing to pay a higher price for food products from sustainable productions, thus demonstrating they are highly sensitivity to and aware of the environmental impact of food products^38^. Similarly, awareness of and trust in sustainable certifications, both domestic and international, influence the behavior of Chinese consumers to a great extent, although other product attributes, such as price, brand reputation, and product quality remain important for consumers’ choices^11^.

Since the literature has indicated that: (1) the assessment of the importance and perception of environmental certifications appears to be product-specific, and to be influenced by the characteristics and geographic affiliation of individuals, (2) in parallel, attention to environmental certifications and changing eating habits appears to be heterogenous across different European and extracontinental countries, and (3) given the gap in the literature on research that simultaneously compares these cross-country aspects by considering consumers’ pro-environmental attitudes toward different products, this research has posed the following research questions: (i) how do environmental concerns influence food consumption? (ii) what is the degree of consumers’ interest in sustainable food certifications? and (iii) how do gender, age, and country of origin differ in their impact on individuals’ food decision-making?

Improving knowledge on how consumers’ consumption and purchasing habits change in relation to climate change through in-depth analysis can help bridge the significant gap between consumers’ interests and the actions they introduce to be more sustainable. The main objective of this study has been to provide an overview of food purchasing frequency, highlighting the changes in food consumption associated with consumers’ concerns about climate change and interest in sustainable food certifications, and to identify the main factors that drive these changes. To do so, consumers’ preferences and perceptions about sustainability certifications were compared considering different foods—such as cheeses, cured meat, pork and beef, fruits and vegetables-, which are often linked to various environmental issues^3,11–13^. The selected types of food are the most commonly consumed food products in the European diet^39–42^. In addition, the collected data of six countries: Italy, France, Germany, Denmark, the USA, and China were compared. This choice of countries was dictated by the need to compare different European and non-European countries that were heterogeneous in terms of culinary tradition, eating styles, individuals’ sensitivity to the issue of sustainability, and food production systems.

## Results

A total of 54% of the surveyed respondents were women, 67% were adults ranging from 18 to 31 years, and 24% had a family composition of 4 components. Gender and age were the main factors that influenced the frequency of food purchases, the effect of climate change on food consumption, and the level of interest in sustainable food certification across countries to a great extent.

### Gender difference in the purchasing habits, interest in sustainable food certifications, and changes in the reported consumption associated with climate change concerns

Table 1 shows that the purchase frequency was higher in U.S. men (*P* ≤ 0.05) for all the food categories (fresh cheeses, aged cheeses, cured meat, pork and beef, fruits and vegetables). In addition, the frequency of purchasing pork and beef was higher in the men population in almost all the countries, except China (*P* ≤ 0.05). The frequency of purchasing aged cheeses and cured meat was higher for Danish, French, German, and U.S. men (*P* ≤ 0.05). On the other hand, Chinese, Danish, German, and Italian women reported higher purchases of vegetables (*P* ≤ 0.05), while German and Italian women showed higher purchases of fruits (*P* ≤ 0.05). Interestingly, the men populations in Denmark, the United States, and France showed a higher interest in the sustainable certifications of cold cuts, while women from Denmark, Germany, and Italy showed higher interest scores for the sustainable certifications of fruits and vegetables (*P* ≤ 0.05). Significantly higher levels of change toward the consumption of cured meat and pork and beef due to climate change were observed in the women population of Denmark, France, Germany, and Italy (*P* ≤ 0.05). Instead, U.S. men showed lower levels of change in the consumption of cheeses, cold cuts, meat, fruits, and vegetables due to climate change (*P* ≤ 0.05).

**Table 1:** The respondents’ demographics and sample characteristics for each studied country

	Country						Total (%)	*Χ*^2^	*P* value
	China (%)	Denmark (%)	France (%)	Germany (%)	Italy (%)	The USA (%)			
Gender									
Women	50	49	51	61	60	51	54	63.290	***
Men	50	51	49	39	40	49	46		
Age									
Adult	67	70	82	65	59	62	67	263.913	***
Elderly	23	13	10	27	31	25	22		
Young	11	17	8	9	10	13	11		
Family composition									
1 component	1	19	17	22	9	14	14	621.423	***
2 components	8	27	20	29	22	23	22		
3 components	51	24	30	28	35	29	33		
4 components	28	22	25	16	29	26	24		
More than 5 components	12	9	9	5	6	9	8		
Buyer									
Single buyer	95	70	84	78	76	83	81	242.347	***
Two buyers	5	30	17	22	24	17	19		

Young = 18–30 years old; Adults = 31–50 years old; Elderly = more than 50 years old. Note: Chi-square test. Significance level: *p* < 0.001 ***.

### Age difference in purchasing behavior, interest in sustainable food certifications, and changes in the reported consumption associated with climate change

Both the young and adult age groups showed a significantly higher frequency of food purchases in all the countries, except in France (Supplementary materials). Adults from Denmark, Germany, and the USA showed a higher frequency of purchasing fresh cheeses, aged cheeses, cured meat, pork, beef, fruits, and vegetables than the elderly (*P* ≤ 0.05). Moreover, the adults from Germany and the USA were more interested in cheeses, cured meat, and sustainable meat certifications than the elderly populations (*P* ≤ 0.05). The young population from Denmark showed a significantly higher interest in all food certification products than the elderly (*P* ≤ 0.05). However, a lower level of change toward consuming cheeses, cured meat, meat, fruits, and vegetables due to climate change was reported for the young Danish, German, and USA consumers than the elderly (*P* ≤ 0.05). (Table 2).

**Table 2:** Gender effect on the food purchasing frequency, levels of interest in sustainable food certifications, and changes in the reported food consumption associated with climate change concerns in the different selected countries

A)																					
	China							Denmark							France						
	Men			Women				Men			Women				Men			Women			
	mean	SD		mean	SD		*P*-value	mean	SD		mean	SD		*P*-value	mean	SD		mean	SD		*P* value
Purchasing frequency																					
Fresh cheeses	4.49	1.58		4.47	1.54		0.8039	4.33	1.39	a	4.33	1.32	b	<0.0001	4.60	1.25	a	4.37	1.19	b	0.0052
Aged cheeses	4.32	1.54		4.33	1.55		0.9786	4.10	1.45	a	4.10	1.46	b	<0.0001	4.43	1.35	a	4.16	1.23	b	0.0000
Cured meat	4.03	1.84		3.93	1.77		0.3801	4.03	1.54	a	4.03	1.56	b	<0.0001	4.34	1.37	a	3.94	1.38	b	<0.0001
Pork	4.92	1.33		4.95	1.29		0.7532	4.41	1.47	a	4.41	1.49	b	<0.0001	4.04	1.61	a	3.62	1.55	b	<0.0001
Beef	4.78	1.25		4.72	1.26		0.4509	4.64	1.35	a	4.64	1.37	b	<0.0001	4.50	1.30	a	4.10	1.33	b	<0.0001
Fruits	5.29	1.31		5.39	1.24		0.2059	5.09	1.14		5.09	0.98		00.2297	5.18	1.13		5.11	1.06		0.3137
Vegetables	5.48	1.46	b	5.65	1.32	a	0.0492	5.12	1.15	b	5.12	0.88	a	<0.0001	5.18	1.08		5.13	1.03		0.4204
Sustainable certifications																					
Cheeses	8.17	1.98		8.18	1.87		0.9738	6.36	2.83	a	6.36	3.04	b	0.0214	6.96	2.48		6.82	2.41		0.4393
Cured meat	7.80	2.18		7.89	2.10		0.5353	6.03	2.94	a	6.03	3.07	b	0.0000	6.08	2.76	a	5.34	3.06	b	0.0000
Meat	8.54	1.60		8.39	1.58		0.1641	7.04	2.58		7.04	2.75		0.9065	7.25	2.27		6.95	2.62		0.0547
Fruits and Vegetables	8.73	1.43		8.69	1.49		0.6968	7.20	2.58	b	7.20	2.49	a	0.0138	7.49	2.13		7.54	2.19		0.6840
Changes in the reported consumption behavior																					
Cheeses	3.60	1.03		3.69	0.96		0.1633	3.30	0.96	a	2.96	0.76	b	<0.0001	3.34	0.91	a	3.16	0.81	b	0.001
Cured meat	3.41	1.04		3.53	1.07		0.1094	3.08	1.09	a	2.66	0.94	b	<0.0001	3.09	1.12	a	2.69	1.08	b	<0.0001
Meat	3.67	0.94		3.74	0.98		0.2366	3.14	1.07	a	2.68	0.98	b	<0.0001	3.17	1.08	a	2.69	1.06	b	<0.0001
Fruits	4.03	0.79		4.12	0.85		0.0798	3.55	0.90		3.48	0.87		0.2254	3.64	0.89	a	3.51	0.88	b	0.0223
Vegetables	4.03	0.80		4.11	0.85		0.1338	3.67	0.94		3.66	0.87		0.6650	3.66	0.87		3.57	0.86		0.0808

B)																					
	Germany							Italy							The USA						
	Men			Women				Men			Women				Men			Women			
	mean	SD		mean	SD		*P* value	mean	SD		mean	SD		*P* value	mean	SD		mean	SD		*P* value
Purchasing frequency																					
Fresh cheeses	4.73	1.23		4.59	1.32		0.0634	4.73	0.88		4.73	1.02		0.6773	4.55	1.62	a	4.02	1.65	b	<0.0001
Aged cheeses	4.38	1.30	a	4.11	1.54	b	0.0010	4.47	0.90		4.43	1.08		0.5092	4.21	1.77	a	3.43	1.82	b	<0.0001
Cured meat	4.64	1.34	a	4.34	1.51	b	0.0000	4.55	0.94		4.46	1.17		0.1307	4.08	1.78	a	3.18	1.80	b	<0.0001
Pork	4.18	1.52	a	3.64	1.78	b	<0.0001	4.47	1.00	a	4.13	1.30	b	<0.0001	4.05	1.90	a	3.33	1.85	b	<0.0001
Beef	4.24	1.49	a	3.80	1.68	b	<0.0001	4.61	0.91	a	4.43	1.19	b	0.0032	4.43	1.73	a	3.88	1.74	b	<0.0001
Fruits	5.08	1.04	b	5.33	1.01	a	<0.0001	5.34	0.93	b	5.47	1.06	a	0.0169	4.88	1.73	a	4.53	1.79	b	0.0019
Vegetables	5.09	1.03	b	5.30	1.01	a	0.0000	5.36	0.90	b	5.47	1.02	a	0.0406	4.85	1.73	a	4.53	1.80	b	0.0034
Sustainable certifications																					
Cheeses	7.10	2.51		7.15	2.69		0.8670	7.36	1.89		7.41	2.21		0.396	7.92	2.44	a	7.00	2.91	b	<0.0001
Cured meat	6.85	2.73		6.60	3.00		0.1365	7.46	1.84		7.41	2.22		0.6847	7.51	2.61	a	6.12	3.25	b	<0.0001
Meat	7.21	2.69		7.27	2.91		0.5401	7.78	1.87		7.83	2.19		0.4376	8.01	2.33	a	7.14	2.89	b	<0.0001
Fruits and Vegetables	7.46	2.40	b	7.99	2.28	a	0.0000	7.71	1.85	b	8.10	1.77	a	0.0000	8.15	2.20	a	7.57	2.61	b	0.0000
Changes in the reported consumption behavior																					
Cheeses	3.33	0.85	a	3.17	1.00	b	0.0041	3.10	0.78		3.05	0.83		0.2985	3.76	1.06	a	3.47	1.00	b	<0.0001
Cured meat	2.99	1.11	a	2.68	1.15	b	<0.0001	2.89	0.89	a	2.75	0.99	b	0.0121	3.40	1.15	a	2.94	1.15	b	<0.0001
Meat	2.97	1.05	a	2.70	1.20	b	0.0000	2.87	0.94	a	2.72	0.99	b	0.0070	3.62	1.06	a	3.29	1.08	b	<0.0001
Fruits	3.54	0.84	b	3.64	0.93	a	0.0383	3.61	0.80		3.67	0.84		0.2033	3.92	0.98	a	3.68	0.95	b	0.0000
Vegetables	3.62	0.80		3.69	0.88		0.0846	3.68	0.77		3.76	0.83		0.0712	3.92	0.94	a	3.74	0.93	b	0.0025

*Sustainable certification Interest Scale*: 10 = Extremely interested, 9 = Very interested; 8 = Quite interested, 7 = Somewhat interested; 6 = Slightly interested; 5 = Neither interested nor uninterested, 4 = Slightly uninterested; 3 = Somewhat uninterested; 2 = Very uninterested, 3 = Somewhat uninterested; 1 = Extremely uninterested.

*Changes in the reported consumption behavior Scale*: 5 = Much more, 4 = A little more, 3 = Same as before 2 = A little less, 1 = Much less.

*Purchasing frequency Scale*: 5 = Much more, 4 = A little more, 3 = Same as before 2 = A little less, 1 = Much less.

Different letters indicate statistical differences related to gender differences obtained using a least significant difference test (*P* < 0.05). *P* values were adjusted using Bonferroni’s method.

### Country of origin, age, and their interaction with the level of interest in sustainable food certifications according to gender

**Fig. 1:**
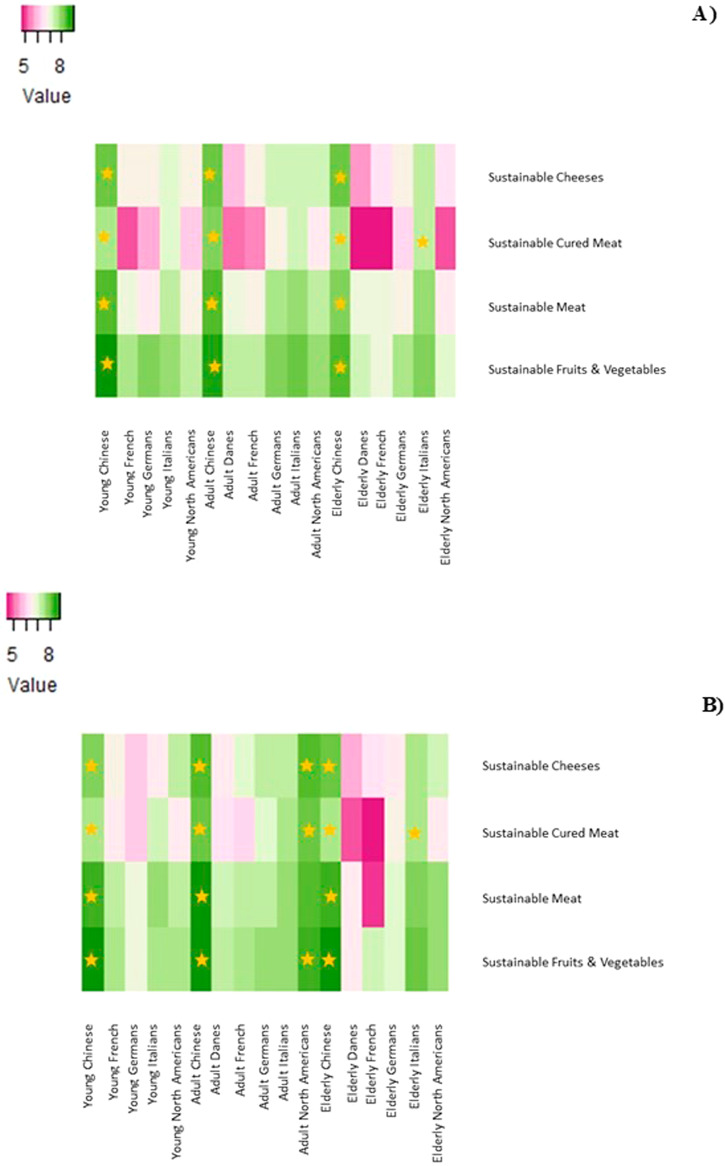
Heatmaps of the levels of interest in sustainable food certifications, and age and country-of-origin of the consumers according to gender. **A** Women and (**B**) Men. The intensity of the colors represents the mean values of the levels of interest in sustainable food certifications and socio-demographics according to gender. The stars represent significantly higher mean values.

High scores of interest in sustainable certifications for cheeses, cured meat, pork and beef, and fruits and vegetables were observed in all the countries, with the exception of a specific part of population in Denmark and France, as shown in Fig. 1 (and in the Supplementary materials). The consumers’ interest in sustainable certifications for food diverged across gender and across the age groups (*P* ≤ 0.05). In fact, the U.S. adult men population reported higher interest scores in the certification of sustainable cheeses, cured meat, and fruits and vegetables than all the other 5 countries (*P* ≤ 0.05). Chinese consumers, both men and women, had higher scores of interest in the certification of sustainable cheeses, cured meat, pork and beef, and fruits and vegetable, regardless of the age group. A stronger age effect was observed for all the sustainable certifications for both women and men adult Danish consumers, who, however, reported a lower interest score (*P* ≤ 0.05). Moreover, the elderly Italian women and men population reported high interest scores for the sustainable certification of cured meat (*P* ≤ 0.05) with respect to the same consumer category of Germany, France, and Denmark.

### Country of origin, age, and their interaction with changes in consumption according to gender

**Fig. 2:**
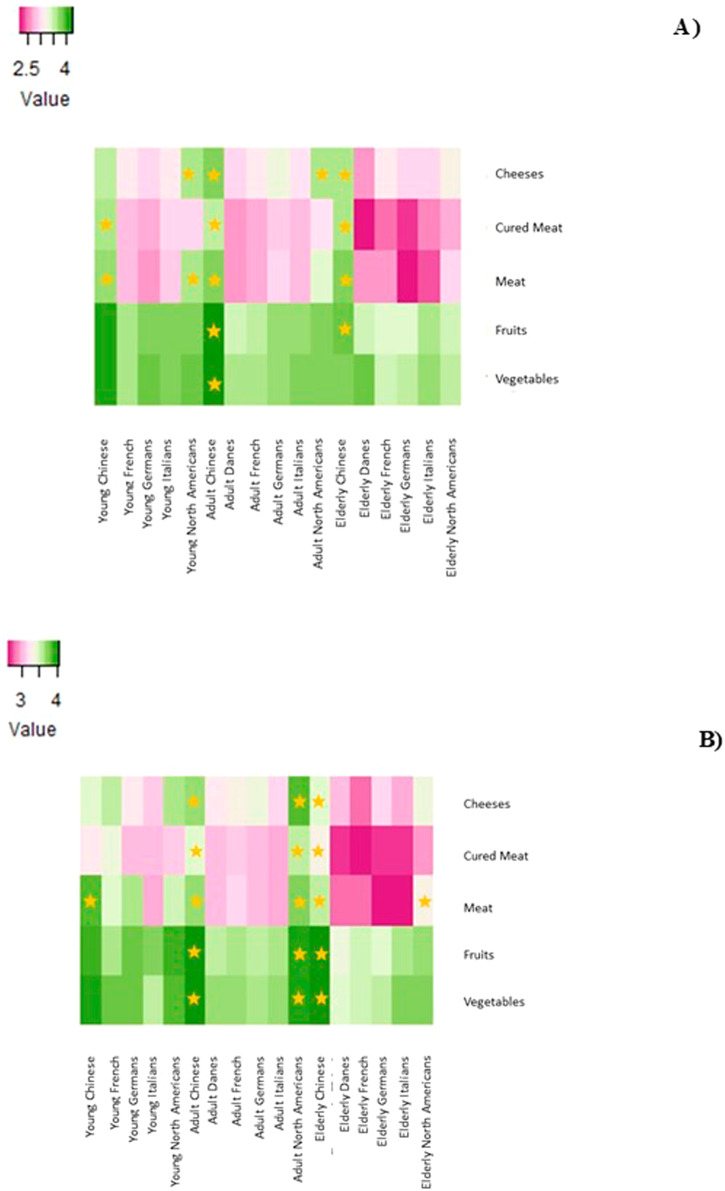
Heatmaps of the changes in the consumers’ consumption associated with climate change concerns, and age and country-of-origin of the consumers according to gender. **A** Women and (**B**) Men. The intensity of the colors represents the mean values of the changes in the consumers’ consumption associated with climate change socio-demographics according to gender. The stars represent significantly higher mean values.

In general, most of the respondents had maintained or reduced their consumption of cheeses, cold cuts, pork and beef, fruits and vegetables in all the considered countries, with the exception of the Chinese population who had increased their intake of fruits and vegetables, regardless of gender or the age group, as shown in Fig. 2 (and in the Supplementary materials, Table 3). The individuals’ consumption changed in relation to the food type across the age groups (*P* ≤ 0.05). Both the women and men Chinese populations had higher consumption scores for cheeses, cured meat, pork and beef, and fruits and vegetables, regardless of the age group, than the other five countries (*P* ≤ 0.05). On the other hand, both the women and men populations in Denmark, in the adult and elderly groups, had a lower consumption of cheeses, cured meat, pork and beef, and fruits (*P* ≤ 0.05). No clear trends of significantly lower food consumption scores were observed in the young population for any food category.

**Table 3:** Age effect on the food purchasing frequency, levels of interest in sustainable food certifications, and changes in the reported food consumption associated with climate change concerns in: (A) China, Denmark, France, and (B) Germany, Italy, and the USA

A)																														
	China										Denmark										France									
	Young			Adults			Elderly				Young			Adults			Elderly				Young			Adults			Elderly			
	mean	SD		mean	SD		mean	SD		*P* value	mean	SD		mean	SD		mean	SD		*P* value	mean	SD		mean	SD		mean	SD		*P* value
Purchasing frequency																														
Fresh cheeses	4.35	1.47	ab	4.59	1.53	a	4.21	1.66	b	0.0043	4.65	1.45	a	4.06	1.35	b	3.77	1.20	b	<0.0001	4.34	1.15		4.52	1.23		4.25	1.21		0.0559
Aged cheeses	4.39	1.45	ab	4.42	1.51	a	4.02	1.64	b	0.0032	4.54	1.47	a	3.76	1.45	b	3.52	1.31	b	<0.0001	4.26	1.22		4.33	1.32		4.03	1.16		0.0750
Cured meat	3.91	1.65	ab	4.15	1.79	a	3.53	1.84	b	0.0000	4.45	1.53	a	3.63	1.59	b	3.42	1.29	b	<0.0001	4.29	1.39		4.16	1.40		3.88	1.29		0.1113
Pork	5.33	1.09	a	5.01	1.25	b	4.53	1.47	c	<0.0001	4.62	1.53	a	3.96	1.54	b	4.17	1.15	b	<0.0001	3.81	1.65		3.87	1.61		3.53	1.38		0.0980
Beef	4.79	1.13	a	4.87	1.21	a	4.38	1.39	b	<0.0001	4.82	1.44	a	4.32	1.39	b	4.46	1.07	ab	0.000	4.29	1.38		4.32	1.33		4.15	1.28		0.4946
Fruits	5.62	1.10	a	5.37	1.23	a	5.11	1.43	b	0.0013	5.19	1.20	a	5.15	1.06	a	4.91	0.89	b	0.022	5.14	1.06		5.15	1.10		5.09	1.10		0.8611
Vegetables	5.90	1.12	a	5.58	1.31	ab	5.38	1.71	b	0.0072	5.18	1.32	ab	5.33	0.97	a	4.96	0.87	b	0.000	5.04	0.97		5.17	1.07		5.13	1.05		0.5038
Sustainable certifications																														
Cheeses	8.05	2.03		8.22	1.92		8.12	1.90		0.5979	6.64	2.85	a	6.14	2.94	ab	5.56	2.96	b	0.0066	6.45	2.69		6.97	2.39		6.54	2.64		0.0646
Cured meat	7.56	2.20	a	7.97	2.16	a	7.61	2.02	a	0.0478	6.51	2.80	a	5.62	3.04	b	4.90	2.98	c	<0.0001	5.49	3.14	ab	5.85	2.91	a	4.74	2.84	b	0.0012
Meat	8.38	1.58		8.52	1.61		8.33	1.54		0.2942	7.29	2.51	a	7.06	2.69	ab	6.55	2.70	b	0.0509	6.91	2.59		7.14	2.45		6.86	2.38		0.4361
Fruits and Vegetables	8.82	1.45		8.74	1.50		8.57	1.34		0.2197	7.49	2.56	a	7.51	2.48	a	6.64	2.72	b	0.0014	7.34	2.40		7.58	2.10		7.12	2.39		0.0956
Changes in the reported consumption behavior																														
Cheeses	3.5	1.04	ab	3.71	0.99	a	3.51	0.99	b	0.0094	3.55	1.14	a	3.06	0.81	b	2.98	0.73	b	<0.0001	3.19	0.96		3.27	0.87		3.12	0.75		0.1829
Cured meat	3.44	1.10		3.49	1.04		3.42	1.09		0.6719	3.41	1.15	a	2.79	0.98	b	2.61	0.96	b	<0.0001	2.9	1.19	a	2.93	1.13	a	2.5	0.92	b	0.0014
Meat	3.78	0.87		3.72	0.95		3.62	1.04		0.2825	3.44	1.20	a	2.80	1.02	b	2.80	0.79	b	<0.0001	3.17	1.21	a	2.94	1.09	a	2.64	0.99	b	0.0064
Fruits	4.01	0.85	a	4.12	0.78	a	3.97	0.91	a	0.0461	3.64	1.05	a	3.51	0.88	ab	3.39	0.64	b	0.0151	3.71	0.97		3.58	0.88		3.41	0.86		0.0797
Vegetables	4.02	0.90	ab	4.14	0.79	a	3.89	0.84	b	0.0000	3.80	1.06	a	3.66	0.88	ab	3.54	0.78	b	0.0184	3.71	0.86		3.62	0.87		3.47	0.83		0.1381

B)																														
	Germany										Italy										The USA									
	Young			Adults			Elderly				Young			Adults			Elderly				Young			Adults			Elderly			
	mean	SD		mean	SD		mean	SD		*P*-value	mean	SD		mean	SD		mean	SD		*P*-value	mean	SD		mean	SD		mean	SD		*P*-value
Purchasing frequency																														
Fresh cheeses	4.50	1.61	ab	4.78	1.27	a	4.37	1.14	b	<0.0001	4.66	1.04		4.76	0.97		4.70	0.92		0.3797	4.49	1.58	a	4.57	1.56	a	3.42	1.64	b	<0.0001
Aged cheeses	4.03	1.59	ab	4.38	1.46	a	3.88	1.34	b	<0.0001	4.55	1.09		4.47	1.03		4.38	0.96		0.1522	3.87	1.91	a	4.15	1.78	a	2.93	1.66	b	<0.0001
Cured meat	4.07	1.79	b	4.61	1.43	a	4.20	1.33	b	<0.0001	4.64	1.18	a	4.54	1.07	a	4.36	1.07	b	0.0102	3.80	1.85	a	3.97	1.81	a	2.64	1.57	b	<0.0001
Pork	3.28	2.04	b	4.14	1.65	a	3.33	1.53	b	<0.0001	4.37	1.32	a	4.36	1.16	a	4.06	1.20	b	0.0000	4.13	1.85	a	3.99	1.86	a	2.65	1.65	b	<0.0001
Beef	3.72	1.69	b	4.31	1.60	a	3.23	1.37	c	<0.0001	4.62	1.19	ab	4.55	1.08	a	4.37	1.07	b	0.0171	4.44	1.51	a	4.43	1.66	a	3.25	1.83	b	<0.0001
Fruits	5.35	0.98	a	5.29	1.05	a	5.03	0.95	b	0.0000	5.41	0.83		5.38	1.06		5.49	0.96		0.2499	5.02	1.44	a	4.96	1.62	a	3.89	2.03	b	<0.0001
Vegetables	5.41	1.00	a	5.28	1.06	a	5.01	0.92	b	<0.0001	5.37	0.92		5.44	0.98		5.41	0.98		0.6543	4.8	1.56	a	4.98	1.63	a	3.88	1.99	b	<0.0001
Sustainable certifications																														
Cheeses	6.91	2.68	ab	7.31	2.55	a	6.77	2.72	b	0.0043	7.02	2.17		7.38	2.03		7.52	2.17		0.0672	7.05	2.83	b	7.82	2.55	a	6.72	2.95	b	<0.0001
Cured meat	5.91	3.09	b	6.92	2.80	a	6.43	2.99	b	0.0000	7.14	2.09		7.42	2.03		7.55	2.14		0.1721	6.34	3.10	b	7.38	2.78	a	5.60	3.23	c	<0.0001
Meat	6.67	3.10	b	7.47	2.67	a	6.91	3.03	b	0.0000	7.60	2.09		7.80	2.02		7.90	2.15		0.4287	7.11	2.93	b	7.90	2.48	a	6.97	2.83	b	<0.0001
Fruits and Vegetables	8.00	2.05	ab	7.88	2.30	a	7.48	2.50	b	0.0193	7.83	1.55		7.91	1.76		8.04	1.98		0.2794	7.51	2.65	b	8.13	2.27	a	7.33	2.63	b	<0.0001
Changes in the reported consumption behavior																														
Cheeses	2.99	1.15	a	3.35	0.98	b	3.03	0.69	b	<0.0001	3.15	0.89	ab	3.10	0.85	a	2.99	0.70	b	0.0204	3.63	1.02	a	3.75	1.06	a	3.27	0.90	b	<0.0001
Cured meat	2.71	1.23	a	2.96	1.17	a	2.44	0.96	b	<0.0001	3.01	1.11	a	2.87	0.96	a	2.61	0.84	b	<0.0001	3.05	1.20	a	3.34	1.19	a	2.78	0.99	b	<0.0001
Meat	2.67	1.21	a	3.01	1.17	a	2.35	0.94	b	<0.0001	2.89	1.07	a	2.90	0.98	a	2.51	0.88	b	<0.0001	3.59	1.06	a	3.58	1.09	a	3.05	0.96	b	<0.0001
Fruits	3.83	0.99	a	3.64	0.91	a	3.43	0.79	b	<0.0001	3.75	0.87		3.65	0.82		3.62	0.80		0.2974	3.83	0.95	a	3.90	0.98	a	3.52	0.91	b	<0.0001
Vegetables	3.83	0.92	a	3.68	0.87	a	3.55	0.76	b	0.0025	3.74	0.85		3.75	0.80		3.69	0.81		0.4965	3.87	1.03	a	3.90	0.93	a	3.63	0.88	b	0.0013

*Sustainable certification Interest Scale*: 10 = Extremely interested, 9 = Very interested; 8 = Quite interested, 7 = Somewhat interested; 6 = Slightly interested; 5 = Neither interested nor uninterested, 4 = Slightly uninterested; 3 = Somewhat uninterested; 2 = Very uninterested, 3 = Somewhat uninterested; 1 = Extremely uninterested.

*Changes in the reported consumption behavior Scale*: 5 = Much more, 4 = A little more, 3 = Same as before 2 = A little less, 1 = Much less.

*Purchasing frequency Scale*: 5 = Much more, 4 = A little more, 3 = Same as before 2 = A little less, 1 = Much less.

Different letters indicate statistical differences related to gender differences obtained using a least significant difference test (*P* < 0.05). *P* values were adjusted using Bonferroni’s method.

### Country of origin, age, and their interaction with food purchasing habits according to gender

**Fig. 3:**
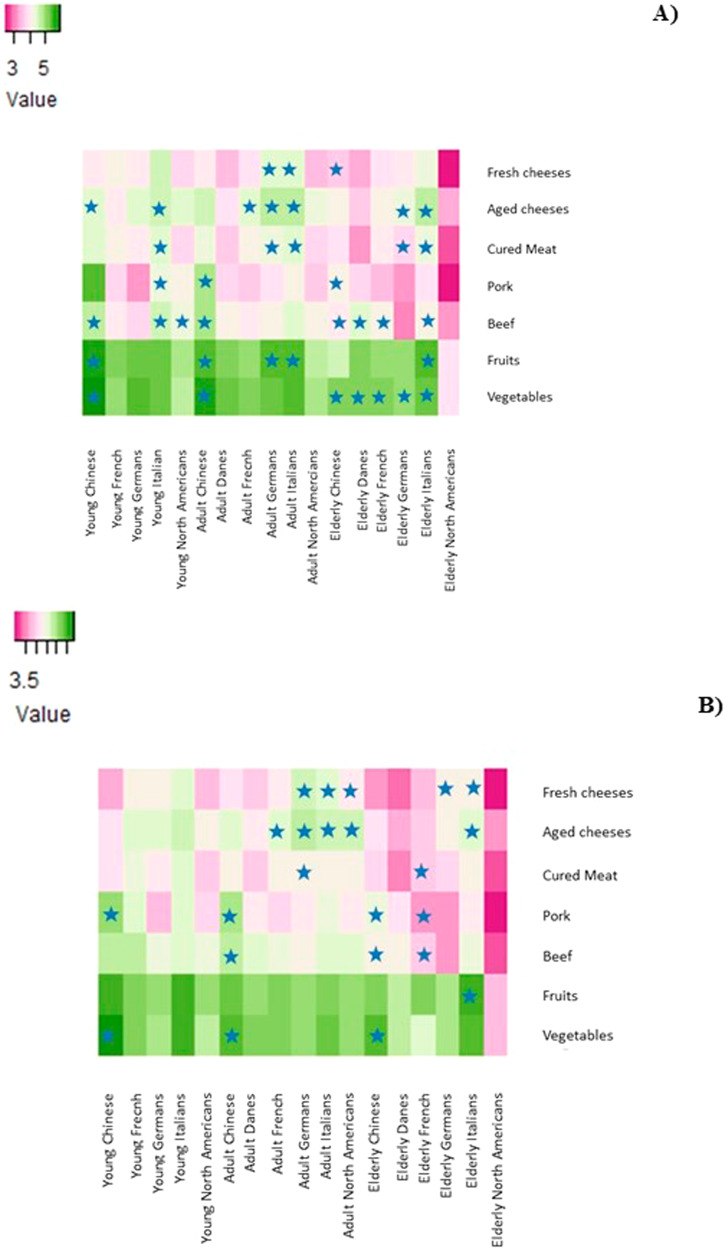
Heatmaps of the food purchase frequency, and age and country-of-origin of the consumers according to gender. **A** Women and (**B**) Men. The intensity of the colors represents the mean values of the food purchasing frequency and socio-demographics according to gender. The stars represent significantly higher mean values.

As shown in Fig. 3, the changes in the consumers’ purchasing habits differed between the age groups (*P* ≤ 0.05) (Supplementary Materials). A higher frequency of purchasing pork, beef, and vegetables was found in the adult and elderly women and men population in China (*P* ≤ 0.05). The Italian elderly women and men population reported a higher frequency of purchasing aged cheeses, beef, and fruits (*P* ≤ 0.05). However, the elderly Italian women reported a significantly higher purchase frequency of cured meats and vegetables, while the men in the same age group reported a higher purchase frequency of fresh cheeses (*P* ≤ 0.05). As for the adult population, Germans and Italians had a higher purchasing frequency of fresh and aged cheeses, The Chinese respondents preferred to purchase pork, beef, and vegetables, while the French preferred to purchase aged cheeses (*P* ≤ 0.05). Interestingly, the adult men population in the United States showed a higher frequency of purchasing fresh and aged cheeses (*P* ≤ 0.05). The differences in the food purchases of young consumers were less significant between countries. The young women population in Italy reported a higher frequency of purchasing aged cheeses, pork, and beef than the other countries (*P* ≤ 0.05). Chinese women reported a higher frequency of purchasing aged cheeses, beef, fruits, and vegetables (*P* ≤ 0.05) than the other countries.

## Discussion

In our study, we have studied the shifts in consumers’ food purchasing habits and their interest in sustainable certifications, and we have reported their changes in consumption in response to climate change across a sample of six countries: our findings reveal that the Chinese consumers declared a more pronounced food consumption and showed a stronger inclination toward sustainable certifications in response to the impacts of climate change. These results are aligned with those of a study on the Chinese population that showed an augmented environmental conciousness^43^. This shift has in part been attributed the heightened environmental awareness and sense of responsibility generated by the Covid-19 pandemic, which has led to an increased reliance on sustainability certifications^44^, particularly in the context of fruits and vegetables^31^.

In general, the consumers tended to perceive health benefits more than achieving a low environmental impact when choosing a healthy and sustainable diet high in fruits and vegetables^20^. Previous research shows that the consumption of a large variety of fruits and vegetables in all the meals of Danish consumers^45^ is influenced by the green policies implemented over the last decade, which have enabled consumers to become more aware of environmental issues and have consequently affected their food choices and lifestyles^46^. This could explain our results on the Danish consumers, who were found to be less interested in sustainable meat certifications. Previous research that showed that government initiatives are crucial in promoting sustainable food choices and limiting food waste supports this general explanation^46,47^. Energy Star, a U.S. government program that promotes energy conservation, has been shown to positively influence consumers’ choices regarding sustainable products^48^.

Other strategies have been implemented to reduce climate change, such as promoting an increased consumption of fruits and vegetables^49^, and raising consumers’ awareness of the importance of the ethical issues that arise from the consumption of meat. Research indicates that the Danish population has increased its intake of plant-based diets, in parallel with a gradual decrease in the consumption of fresh and cured meat^32,50^. Consumers who adopt environmentally friendly behaviors generally report a reduction in the frequency of meat consumption^20^. According to Silva et al., consumers consider sustainability to be a crucial factor in food choices. Indeed, such concepts as “zero-kilometer” and a focus on consuming locally sourced and seasonal products have gained popularity among these consumers^51^. However, according to ref. ^18^ consumers are not fully aware of the actual impact of livestock production, and not all countries are developing green energy policies, while discussions are conducted on a macro level and have not yet reached consumers^52^. Existing research suggests that although Italian consumers are aware of the impact of fresh meat on the planet, the association with the consumption of cured meats, such as salami and ham, has not been as evident as the concerns raised for the consumption of fresh meat^20^. In addition, the close link between culinary culture, gastronomic variety, local history, and local identity regarding the use of distinct ingredients from each country, such as salami and ham in Italy, provides a clear understanding of the consumption behavior of different countries^53^. Therefore, future research is needed to explore how the history, geography, climate, culture, and economy of a country can influence changes in food consumption associated with climate change across different regions and countries.

A key question that emerges from our observational study is: why do Chinese consumers rely on sustainable certifications more than in other countries? In China, the “Green Food” certification, which covers food safety and environmental friendliness, has helped give a positive signal of a sustainable product to Chinese^54^ by reducing skepticism caused by the numerous counterfeits detected in their country^44^. However, a preference for green labels is also highly dependent on gender and age. Existing research indicates that green labels are preferred by men, while organic labels are preferred by women^55^. The clear divergence and lack of clarity of sustainable certifications may confuse consumers and ultimately affect the discrepancies found between gender^56^. As far as the age effect is concerned, a recent study has found that eco-labels are more likely to be read by adults^31^. However, according to ref. ^15^, young Northern European populations are the most willing to change their eating habits due to climate change, and, along with Europeans, the U.S. population is willing to reduce meat consumption^57^. Future studies could explore a specific supply chain and simultaneously conduct economic analyses, for example, to examine respondents’ self-reported willingness to pay and in function of their financial conditions.

The environmental impact of consumers’ food consumption estimates can provide evidence that can in turn be used to support policies and programs that promote sustainable healthy diets. In this context, environmental and social responsibility are increasingly influencing consumers’ food choices^58^. From the consumers’ perspective, more programs dedicated to raising the awareness of the environmental concerns of the whole food supply chain, including the production, storage, delivery, and retailing of food to reach consumers are needed to facilitate a transition toward more sustainable consumption. In terms of the food policy framework, further support for food quality schemes for producers, processors, retailers, consumers, and policymakers is required to create a base on the sustainability of the entire food production chain and on the consequent environmental impact.

The results of this study are robust as far as assumptions about purchase frequency, interest level, and changes in food consumption associated with climate concerns are concerned. Nevertheless, our method and data are subject to several limitations, including the exclusion of a ‘nonbinary’ choice in the survey and the questionnaire was not back-translated.

In general, research on motivations for and against participation may be a starting point to overcome recruitment difficulties. Memory bias and self-selection bias are two of the other limitations of online surveys. However, the fact that the survey was conducted online, thereby allowing participants to respond at their own pace, is one of the strengths of the survey, and the high response, that is, of about 1300 respondents from each country is another. Honest responses were encouraged by conducting the survey online, thereby reducing social desirability. Each participant was able to complete the survey in a private setting, thus removing themselves from the possible influence or judgment of third parties on the responses.

Overall, our analysis indicates that the considered consumers’ purchasing and consumption of a range of food products underscore a lack of awareness of the environmental implications of dietary choices on climate change, as it appears challenging for some consumers to alter their eating habits. We also show that the frequently debated opinion of using sustainable certifications to reduce climate change does not seem the best sustainability strategy for all consumers. Indeed, deeply rooted gastronomic traditions in food culture create a significant challenge for consumers to reduce their dependence on products of animal origin.

## Methods

Data were collected online from January to June 2022 by the Teleperformance company in consultancy business, data collection, and the analysis of customers’ experiences. The Computer Assisted Web Interviewing (CAWI) was used as data collection methodology in order to select a sample of consumers equally distributed over the chosen areas (nearly 1300 individuals per country). Initially, the questionnaire was drawn up in Italian and was pre-tested by experts in consumer science. After their approval, the questionnaire (Supplementary Table 1) was translated into English, German, French, Chinese, and Danish by native speakers to allow the respondents to fully understand the text and to enable maximum efficiency of the answers. The translated surveys were pre-tested by a minimum of 10 subjects with residence in each of the countries under study who were unrelated to the project to identify problems related to the phrasing of the questions, omissions, and other difficulties experienced by respondents, as has been done in other studies^59,60^. Minor modifications were made related to the phrasing of the questions and response options. These pre-tests were used to obtain feedback for the researchers, and the questionnaire was adjusted accordingly. The online survey was anonymous, and the respondents electronically signed an informed consent form before participating in the survey and after having read a disclosure sheet that described the project and survey aims. The study protocol was approved by the Ethical Committee of the University of Turin (Ref - GD/14849/2020), and all the researchers participated in the development of the questionnaire and approved its content and dissemination methods. The research was conducted according to the Declaration of Helsinki guidelines^61^. One of the aims of this work was to examine how such factors as gender, age, and country influence the frequency of purchasing food, the effect of climate change on food consumption, and the level of interest in sustainable food certification.

### Participants

A multi-country study was developed using a quota sample of 6500 respondents recruited from an online panel from Italy, Germany, France, the USA, China, and Denmark. The selected countries were chosen for several reasons. First, the chosen six countries represent a diversified target of consumers and climate change impacts. Second, the countries were selected in order to include both high climate performance countries (i.e., Denmark, France, and Germany) and lower performance countries, according to Climate Change performance Index 2024 (i.e., Italy, China, and the USA)^62^. Moreover, each country has unique food habits and attitudes; Italy, for example, has a strong and long-standing gastronomic tradition, while others, like the USA, prefer a more traditional cuisine^63^. The questionnaire was distributed to the panelists through the survey link and the participants were not paid for their participation. Resorting to quota sampling ensured that the sample reflected the adult population, in terms of age, gender, and race. The rates of completion of the survey for each country are reported in Supplementary Table 2. The inclusion criteria of the participants were: (i) individuals who agreed to participate and who gave their consent for data usage in the first question of the questionnaire; (ii) the individuals had to be over 18 years of age; (iii) the individuals had to come from 1 of the following countries: Italy, Germany, France, the USA, China or Denmark.

### Survey design and questionnaire

A cross-sectional questionnaire was developed in four sections. In particular, the questionnaire included the following measures:

- socio-demographic characteristics (gender, age, family composition);
- food purchasing frequency: this self-constructed scale composed by seven items (*fresh cheeses, aged cheeses, cured meat, pork, beef, fruits, vegetables)* was adapted^30,36,40,64,65^ to measure the actual food purchasing frequency of the different foods using a 7-point internal scale (ranging from 1 = never to 7 = more than 5 times per week). Cronbach’s Alpha was equal to 0.94;
- sustainable certifications: this scale was composed of four items (*cheeses, cured meat, meat, fruits, and vegetables) and was developed* to rate the level of interest of individuals in the sustainable certification, using a 10-point Likert scale (ranging from 1 = not at all important to 10 = extremely important)^40,65,66^. Cronbach’s Alpha was equal to 0.86.
- Changes in the reported consumption behavior: this scale measures the level of change in their consumption for five items *(cheeses, cured meat, fresh meat, fruits, vegetables)* in function of their concern about the climate change issue, using a 5-point interval scale (ranging from 1 = much less to 5 = much more)^64,67^. Cronbach’s Alpha was equal to 0.90.

The choice of using 5-, 7- and 10-point scales, although unconventional, was made to obtain responses with different levels of detail according to the proposed scales^68,69^. The use of scales with a specific number of points is a common and even recommended practice in some fields and disciplines. Moreover, it is common practice to opt for a scale with a different number of points to highlight the relevance or importance of a question and/or to emphasize particular questions or concepts. In fact, the 10-point scale was used to achieve a greater precision in the responses and to capture more subtle nuances in the participants’ opinions. It was used in the developed questions to measure the level of interest of the participants in sustainable certifications.

The other scales used 5- and 7-points, but these scales do not show any statistically significant differences in terms of normality and reliability^70^. The choice of the 5-point scale to measure the willingness of a participant to change their consumption habits was made to be more concrete and less misleading in obtaining a response that analyzes future prospects for sustainable consumption.

The respondents were categorized according to age: (i) young: 18–30, (ii) adults: 31–50, and (iii) elderly: over 51, and according to gender (i) women and (ii) men.

### Statistical analysis

A comparison of the mean scores was made to assess the associations between the frequency of food purchases (interval variables, seven-point scale) for different food categories, the levels of interest in sustainable certifications for each food product (interval variables, ten-point scale), and the level of change in food consumption due to climate change concerns (interval variables, five-point scale), and it was carried out using generalized linear mixed-effect models (*glmm*). Mixed models were chosen because of their ability to capture both fixed effects (Gender: women and men; Age: young, adult, and elderly; and Country: Italy, Germany, France, Denmark, China, and the USA) and random effects (number of subjects, *n* = 6500). The *P* values were adjusted using Bonferroni’s method and when the mixed model revealed significant differences (*P* < 0.05), the least significant difference test was applied. The mixed models were built and evaluated according to Crawley, (2012), using version 3.3.2 of R for Windows. Power calculation of the sample size was used to ensure a significance level = 0.05 and f values = 0.4, using the *pwr* function (power = 1). Spearman’s rank correlation coefficient was obtained, as a measure of the association between changes in food consumption due to climate change and interest in sustainable food certifications, using the psych function, and plotted using the *corrplot* package of R for Windows.

### Reporting summary

Further information on research design is available in the Nature Research Reporting Summary linked to this article.

### Supplementary information

Reporting Summary

Suppementary Material

## Data Availability

The datasets used and/or analyzed during the current study are available from the corresponding author upon request.

## References

[1] Mansoor M, Awan TM, Paracha OS (2022). Sustainable buying behaviour: an interplay of consumers’ engagement in sustainable consumption and social norms. Int. Soc. Sci. J..

[2] Zeng Z, Zhong W, Naz S (2023). Can environmental knowledge and risk perception make a difference? The role of environmental concern and pro-environmental behavior in fostering sustainable consumption behavior. Sustainability.

[3] Nguyen, N. & Mogaji, E. A theoretical framework for the influence of green marketing communication on consumer behaviour in emerging economies. 10.1007/978-3-030-82572-0_11 (2021).

[4] de Boer J (2003). Sustainability labelling schemes: the logic of their claims and their functions for stakeholders. Bus. Strategy Environ..

[5] Leach AM (2016). Environmental impact food labels combining carbon, nitrogen, and water footprints. Food Policy.

[6] Credence Attributes, Consumers Trust and Sensory Expectations in Modern Food Market: Is there a Need to Redefine their Role? *International Journal on Food System Dynamics*10.22004/ag.econ.277723 (2018).

[7] Fernqvist F, Ekelund L (2014). Credence and the effect on consumer liking of food—a review. Food Qual. Prefer..

[8] Huo H (2023). The effect of credence attributes on willingness to pay a premium for organic food: a moderated mediation model of attitudes and uncertainty. Front. Psychol..

[9] Valor, C., Carrero Bosch, I. & Redondo, R. The influence of knowledge and motivation on sustainable label use. *J. Agric. Environ. Ethics* **27**, 591–607 (2013).

[10] Grimmer M, Woolley M (2014). Green marketing messages and consumers’ purchase intentions: promoting personal versus environmental benefits. J. Mark. Commun..

[11] Smith P (2013). How much land-based greenhouse gas mitigation can be achieved without compromising food security and environmental goals?. Glob. Chang Biol..

[12] Lee RP, Meyer B, Huang Q, Voss R (2020). Sustainable waste management for zero waste cities in China: potential, challenges and opportunities. Clean. Energy.

[13] Hornsey MJ, Harris EA, Bain PG, Fielding KS (2016). Meta-analyses of the determinants and outcomes of belief in climate change. Nat. Clim. Chang..

[14] Pong V (2021). Global versus local framing of the issue of food waste: The role of identification With All Humanity and the implications for climate change communication. Asian J. Soc. Psychol..

[15] Richards CE, Lupton RC, Allwood JM (2021). Re-framing the threat of global warming: an empirical causal loop diagram of climate change, food insecurity and societal collapse. Clim. Chang..

[16] Rudel, T. The variable paths to sustainable intensification in agriculture. *Reg. Environ. Chang.* **20**, 126 (2020).

[17] Owino, V. et al. The impact of climate change on food systems, diet quality, nutrition, and health outcomes: a narrative review. *Front. Clim.* **4**, 941842 (2022).

[18] van Bussel LM, Kuijsten A, Mars M, van ‘t Veer P (2022). Consumers’ perceptions on food-related sustainability: a systematic review. J. Clean. Prod..

[19] Abbass K (2022). A review of the global climate change impacts, adaptation, and sustainable mitigation measures. Environ. Sci. Pollut. Res..

[20] Bimbo F (2023). Climate change-aware individuals and their meat consumption: evidence from Italy. Sustain. Prod. Consum..

[21] Piracci G, Casini L, Contini C, Stancu C, Lähteenmäki L (2023). Identifying key attributes in sustainable food choices: an analysis using the food values framework. J. Clean. Prod..

[22] Ferrari M (2020). Could dietary goals and climate change mitigation be achieved through optimized diet? The experience of modeling the national food consumption data in Italy. Front Nutr..

[23] Farchi S, De Sario M, Lapucci E, Davoli M, Michelozzi P (2017). Meat consumption reduction in Italian regions: health co-benefits and decreases in GHG emissions. PLoS One.

[24] Zucali M, Tamburini A, Sandrucci A, Bava L (2017). Global warming and mitigation potential of milk and meat production in Lombardy (Italy). J. Clean. Prod..

[25] Poore J, Nemecek T (2018). Reducing food’s environmental impacts through producers and consumers. Science.

[26] Thøgersen J, Ölander F (2002). Human values and the emergence of a sustainable consumption pattern: A panel study. J. Econ. Psychol..

[27] Juhl HJ, Fenger MHJ, Thøgersen J (2017). Will the consistent organic food consumer step forward? An empirical analysis. J. Consum. Res..

[28] Zhu Y, Wang Z, Zhu X (2023). New reflections on food security and land use strategies based on the evolution of Chinese dietary patterns. Land Use Policy.

[29] Singpai, B. & Wu, D. Measure of China’s environmental economic efficiency. In *Proc. 4th IEEE International Conference on Cybernetics (Cybconf)* 1–7. 10.1109/Cybconf47073.2019.9436628 (2019).

[30] Merlino VM (2022). Are local dairy products better? Using principal component analysis to investigate consumers’ perception towards quality, sustainability, and market availability. Animals.

[31] Li S, Kallas Z, Rahmani D (2022). Did the COVID-19 lockdown affect consumers’ sustainable behaviour in food purchasing and consumption in China?. Food Control.

[32] Lassen AD, Christensen LM, Trolle E (2020). Development of a danish adapted healthy plant-based diet based on the EAT-lancet reference diet. Nutrients.

[33] Yadav R, Pathak GS (2017). Determinants of consumers’ green purchase behavior in a developing nation: applying and extending the theory of planned behavior. Ecol. Econ..

[34] Aprile MC, Caputo V, Nayga RM (2012). Consumers’ valuation of food quality labels: the case of the European geographic indication and organic farming labels. Int. J. Consum. Stud..

[35] Lamonaca E, Cafarelli B, Calculli C, Tricase C (2022). Consumer perception of attributes of organic food in Italy: a CUB model study. Heliyon.

[36] Merlino VM (2023). The role of socio-demographic variables and buying habits in determining milk purchasers’ preferences and choices. Front. Nutr..

[37] Sparacino A, Merlino VM, Blanc S, Borra D, Massaglia S (2022). A choice experiment model for honey attributes: Italian consumer preferences and socio-demographic profiles. Nutrients.

[38] Lanfranchi M, Schimmenti E, Campolo MG, Giannetto C (2019). The willingness to pay of Sicilian consumers for a wine obtained with sustainable production method: An estimate through an ordered probit sample-selection model. Wine Econ. Policy.

[39] Hargreaves SM, Raposo A, Saraiva A, Zandonadi RP (2021). Vegetarian diet: an overview through the perspective of quality of life domains. Int. J. Environ. Res. Public Health.

[40] Sanchez-Sabate R, Sabaté J (2019). Consumer attitudes towards environmental concerns of meat consumption: a systematic review. Int. J. Environ. Res. Public Health.

[41] Serra-Majem L (2020). Updating the Mediterranean diet pyramid towards sustainability: focus on environmental concerns. Int J. Environ. Res. Public Health.

[42] Verduna T, Blanc S, Merlino VM, Cornale P, Battaglini LM (2020). Sustainability of four dairy farming scenarios in an Alpine environment: the Case Study of Toma di Lanzo cheese. Front. Vet. Sci..

[43] Ji Z (2017). Comparing cancer information needs for consumers in the US and China. Stud. Health Technol. Inform..

[44] Xu H, Xiao M, Zeng J, Hao H (2022). Green-labelled rice versus conventional rice: perception and emotion of chinese consumers based on review mining. Foods.

[45] Rekhy R, McConchie R (2014). Promoting consumption of fruit and vegetables for better health. Have campaigns delivered on the goals?. Appetite.

[46] Danish, Ulucak R (2020). The pathway toward pollution mitigation: does institutional quality make a difference?. Bus. Strategy Environ..

[47] Wentz, J. A. & Gerrard, M. Persistent regulations: a detailed assessment of the trump administration’s efforts to repeal federal climate protections. 10.7916/d8-3dcs-3h21 (2019).

[48] Siegrist M, Stampfli N, Kastenholz H (2008). Consumers’ willingness to buy functional foods. the influence of carrier, benefit and trust. Appetite.

[49] *Fruit and Vegetable Consumption by Low-Income Americans: Would a Price Reduction Make a Difference?*10.22004/ag.econ.55835 (2009).

[50] Mithril C (2012). Guidelines for the new Nordic diet. Public Health Nutr..

[51] Silva ME, Sousa-Filho JMD, Yamim AP, Diógenes AP (2020). Exploring nuances of green skepticism in different economies. MIP.

[52] Mazzocchi C, Orsi L, Zilia F, Costantini M, Bacenetti J (2022). Consumer awareness of sustainable supply chains: a choice experiment on Parma ham PDO. Sci. Total Environ..

[53] Contini C (2016). Why do we buy traditional foods?. J. Food Prod. Mark..

[54] Li S, Lopez RA, Zhu C, Liu Y (2023). Consumer preferences for sustainably produced ultra-high-temperature milk in China. J. Dairy Sci..

[55] McCarthy B, Liu H-B, Chen T (2016). Innovations in the agro-food system: adoption of certified organic food and green food by Chinese consumers. Br. Food J..

[56] Aguilar FX, Cai Z (2010). Conjoint effect of environmental labeling, disclosure of forest of origin and price on consumer preferences for wood products in the US and UK. Ecol. Econ..

[57] Ritchie, H., Rosado, P. & Roser, M. Environmental impacts of food production. *Our World in Data* (2022).

[58] Neufeld LM (2022). Food choice in transition: adolescent autonomy, agency, and the food environment. Lancet.

[59] Mitchell KR, Ploubidis GB, Datta J, Wellings K (2012). The Natsal-SF: a validated measure of sexual function for use in community surveys. Eur. J. Epidemiol..

[60] Rodrigues P, Sousa A, Fetscherin M, Borges AP (2024). Exploring masstige brands’ antecedents and outcomes. Int. J. Consum. Stud..

[61] World Medical Association. World Medical Association Declaration of Helsinki: ethical principles for medical research involving human subjects. *Bull. World Health Org*. **79**, 373 (2013). PMC2566407 11357217

[62] Ranking | Climate Change Performance Index. https://ccpi.org/ranking/ (2023).

[63] Menozzi D, Sogari G, Veneziani M, Simoni E, Mora C (2017). Eating novel foods: an application of the Theory of Planned Behaviour to predict the consumption of an insect-based product. Food Qual. Preference.

[64] Sanchez-Sabate R, Badilla-Briones Y, Sabaté J (2019). Understanding attitudes towards reducing meat consumption for environmental reasons. a qualitative synthesis review. Sustainability.

[65] Cerri J, Testa F, Rizzi F (2018). The more I care, the less I will listen to you: how information, environmental concern and ethical production influence consumers’ attitudes and the purchasing of sustainable products. J. Clean. Prod..

[66] Thompson DW, Anderson RC, Hansen EN, Kahle LR (2010). Green segmentation and environmental certification: insights from forest products. Bus. Strat Env.

[67] Nocella G, Romano D, Stefani G (2014). Consumers’ attitudes, trust and willingness to pay for food information. Int. J. Consum. Stud..

[68] Altuna, O. K. & Müge Arslan, F. Impact of the number of scale points on data characteristics and respondents’ evaluations: An experimental design approach using 5-point and 7-point Likert-type scales. *İstanbul Üniversitesi Siyasal Bilgiler Fakültesi Dergisi* **55**, 1–20 (2016).

[69] Russo GM, Tomei PA, Serra B, Mello S (2021). Differences in the use of 5-or 7-point likert scale: an application in food safety culture. Organ. Cult..

[70] Taherdoost, H. What is the best response scale for survey and questionnaire design; review of different lengths of rating scale/attitude scale/Likert scale. *Hamed Taherdoost* **8**, 1–10 (2019).

