# Supplementary material for: Socio-demographic and cross-country differences in attention to sustainable certifications and changes in food consumption
Source: NPJ Sci Food. 2024 Jun 1;8:31. doi: 10.1038/s41538-024-00274-x (PMC11144225; doi:10.1038/s41538-024-00274-x)
Supplement: Supplementary file 1 — Reporting Summary [file 41538_2024_274_MOESM1_ESM.pdf]

nature portfolio

Corresponding author(s): *Double-anonymous peer review submissions: write DAPR and your manuscript number here instead of author names.*

Last updated by author(s): *YYYY-MM-DD*

Reporting Summary

Nature Portfolio wishes to improve the reproducibility of the work that we publish. This form provides structure for consistency and transparency in reporting. For further information on Nature Portfolio policies, see our Editorial Policies and the Editorial Policy Checklist.

Statistics

For all statistical analyses, confirm that the following items are present in the figure legend, table legend, main text, or Methods section.

n/a	Confirmed	
☐	☒	The exact sample size (*n*) for each experimental group/condition, given as a discrete number and unit of measurement
☐	☒	A statement on whether measurements were taken from distinct samples or whether the same sample was measured repeatedly
☐	☒	The statistical test(s) used AND whether they are one- or two-sided<br>*Only common tests should be described solely by name; describe more complex techniques in the Methods section.*
☐	☒	A description of all covariates tested
☐	☒	A description of any assumptions or corrections, such as tests of normality and adjustment for multiple comparisons
☐	☒	A full description of the statistical parameters including central tendency (e.g. means) or other basic estimates (e.g. regression coefficient) AND variation (e.g. standard deviation) or associated estimates of uncertainty (e.g. confidence intervals)
☐	☒	For null hypothesis testing, the test statistic (e.g. *F*, *t*, *r*) with confidence intervals, effect sizes, degrees of freedom and *P* value noted<br>*Give P values as exact values whenever suitable.*
☒	☐	For Bayesian analysis, information on the choice of priors and Markov chain Monte Carlo settings
☒	☐	For hierarchical and complex designs, identification of the appropriate level for tests and full reporting of outcomes
☐	☒	Estimates of effect sizes (e.g. Cohen's *d*, Pearson's *r*), indicating how they were calculated

Our web collection on statistics for biologists contains articles on many of the points above.

Software and code

Policy information about availability of computer code

Data collection	NO SOFWARE WAS USED
Data analysis	Version 3.3.2 of R for Windows

For manuscripts utilizing custom algorithms or software that are central to the research but not yet described in published literature, software must be made available to editors and reviewers. We strongly encourage code deposition in a community repository (e.g. GitHub). See the Nature Portfolio guidelines for submitting code & software for further information.

Data

Policy information about availability of data

All manuscripts must include a data availability statement. This statement should provide the following information, where applicable:

- Accession codes, unique identifiers, or web links for publicly available datasets
- A description of any restrictions on data availability
- For clinical datasets or third party data, please ensure that the statement adheres to our policy

The datasets used and/or analyzed during the current study are available from the corresponding author upon reasonable request

Research involving human participants, their data, or biological material

Policy information about studies with human participants or human data. See also policy information about sex, gender (identity/presentation), and sexual orientation and race, ethnicity and racism.

Reporting on sex and gender	The survey was anonymous. Gender was collected and used in the statistical analysis to understand its fixed effect on the frequency of food purchases (for different food categories, the levels of interest in sustainable certifications for each food product and the level of change in food consumption due to climate change concerns.
Reporting on race, ethnicity, or other socially relevant groupings	n/a
Population characteristics	see above
Recruitment	Data were collected online from January to June 2022 by a specializes company in consultancy business, data collection, and the analysis of customers' experiences. The Computer Assisted Web Interviewing (CAWI) was used as data collection methodology in order to select a sample of consumers equally distributed over the chosen areas
Ethics oversight	n/a (The survey was anonymous and no sensitive data was collected)

Note that full information on the approval of the study protocol must also be provided in the manuscript.

Field-specific reporting

Please select the one below that is the best fit for your research. If you are not sure, read the appropriate sections before making your selection.

☐ Life sciences ☒ Behavioural & social sciences ☐ Ecological, evolutionary & environmental sciences

For a reference copy of the document with all sections, see nature.com/documents/nr-reporting-summary-flat.pdf

Behavioural & social sciences study design

All studies must disclose on these points even when the disclosure is negative.

Study description	A multi-country study was developed using a quota sample of 6,500 respondents recruited from an online-panel from Italy, Germany, France, the USA, China, and Denmark. A cross-sectional questionnaire was developed in four sections: 1) socio-demographics; 2) food purchasing frequency; 3) sustainable certifications; and 4) climatic impact.A comparison of the mean scores was made to assess the associations between the frequency of food purchases (interval variables, seven-point scale) for different food categories, the levels of interest in sustainable certifications for each food product (interval variables, ten-point scale) and the level of change in food consumption due to climate change concerns (interval variables, five-point scale), and it was carried out using generalized linear mixed-effect models (glmm). Mixed models were chosen because of their ability to capture both fixed effects (Gender: women and men; Age: young, adult and elderly; and Country: Italy, Germany, France, Denmark, China, and the USA) and random effects (number of subjects, n= 6 500).
Research sample	A total of 54% of the surveyed respondents were female, 67 % were adults ranging from 18 to 31 years, and 24% had a family composition of 4 components. A multi-country study was developed using a quota sample of 6,500 respondents recruited from an online-panel from Italy, Germany, France, the USA, China, and Denmark nearly 1,300 individuals per country). This choice of countries was dictated by the need to compare different European and non-European countries that were heterogeneous in terms of culinary tradition, eating styles, individuals' sensitivity to the issue of sustainability, and food production systems.
Sampling strategy	This choice of countries was dictated by the need to compare different European and non-European countries that were heterogeneous in terms of culinary tradition, eating styles, individuals' sensitivity to the issue of sustainability, and food production systems. The Computer Assisted Web Interviewing (CAWI) was used as data collection methodology in order to select a sample of consumers equally distributed over the chosen areas (nearly 1,300 individuals per country).
Data collection	online survey was administred by specializes company in consultancy business, data collection, and the analysis of customers' experiences
Timing	from January to June 2022
Data exclusions	The inclusion criteria of the participants were: i) individuals who agreed to participate and who gave their consent for data usage in the first question of the questionnaire; ii) the individuals had to be over 18 years of age; iii) the individuals had to come from 1 of the following countries: Italy, Germany, France, the USA, China or Denmark.
Non-participation	mean responses rate=42%
Randomization	no groups

Reporting for specific materials, systems and methods

We require information from authors about some types of materials, experimental systems and methods used in many studies. Here, indicate whether each material, system or method listed is relevant to your study. If you are not sure if a list item applies to your research, read the appropriate section before selecting a response.

Materials & experimental systems

n/a	Involved in the study
☒	☐ Antibodies
☒	☐ Eukaryotic cell lines
☒	☐ Palaeontology and archaeology
☒	☐ Animals and other organisms
☒	☐ Clinical data
☒	☐ Dual use research of concern
☒	☐ Plants

Methods

n/a	Involved in the study
☒	☐ ChIP-seq
☒	☐ Flow cytometry
☒	☐ MRI-based neuroimaging

Plants

Seed stocks

Report on the source of all seed stocks or other plant material used. If applicable, state the seed stock centre and catalogue number. If plant specimens were collected from the field, describe the collection location, date and sampling procedures.

Novel plant genotypes

Describe the methods by which all novel plant genotypes were produced. This includes those generated by transgenic approaches, gene editing, chemical/radiation-based mutagenesis and hybridization. For transgenic lines, describe the transformation method, the number of independent lines analyzed and the generation upon which experiments were performed. For gene-edited lines, describe the editor used, the endogenous sequence targeted for editing, the targeting guide RNA sequence (if applicable) and how the editor was applied.

Authentication

Describe any authentication procedures for each seed stock used or novel genotype generated. Describe any experiments used to assess the effect of a mutation and, where applicable, how potential secondary effects (e.g. second site T-DNA insertions, mosiacism, off-target gene editing) were examined.
