# Supplementary material for: Socio-demographic and cross-country differences in attention to sustainable certifications and changes in food consumption
Source: NPJ Sci Food. 2024 Jun 1;8:31. doi: 10.1038/s41538-024-00274-x (PMC11144225; doi:10.1038/s41538-024-00274-x)
Supplement: Supplementary file 2 — Suppementary Material [file 41538_2024_274_MOESM2_ESM.pdf]

Supplementary Table

Supplementary Table 1. Measured items

Supplementary Table 2. Survey Response Rates

Supplementary Table 3. Country difference in the levels of interest in sustainable cheeses, cured meat, meat and fruits and vegetables certifications between China, Denmark, France, Germany, Italy and the USA according to gender and age

Supplementary Table 3. Country difference in the changes in reported consumption of cheeses, cured meat, meat and fruits and vegetables associated with climate change between China, Denmark, France, Germany, Italy and the USA according to gender **A)** Men **B)** Women and age group.

Supplementary Table 4. Country difference in the purchasing frequency of fresh cheeses, aged cheeses, cured meat, pork, beef, fruits and vegetables between China, Denmark, France, Germany, Italy and the USA according to gender: **A)** Men **B)** Women and age group.

Supplementary Table 1

Measure	Items
**Purchasing frequency**	How often do you purchase each of the following products from the European Union?<br>1. Cheeses<br>2. Cured meat<br>3. Meat<br>4. Fruits<br>5. Vegetables<br><br>Interval scale (1= Never to 7= more than 5 timer *per* week)
**Climate change impact**	Compared to the food categories that you find below, please indicate if your consumption is changing and how much has changed because of climate change.<br>1. Cheeses<br>2. Cured meat<br>3. Meat<br>4. Fruits<br>5. Vegetables<br><br>Interval scale (1= Much less to 5= Much more)
**Sustainable certification**	How important is it for you a certified or guaranteed sustainable agri-food products in the following categories?<br>1. Cheeses<br>2. Cured meat<br>3. Meat<br>4. Fruits and vegetables<br><br>Interval scale (1= not important at all to 10= extremely important)

Source (s): Authors' compilation

Supplementary Table 2

Country	Respondents	Non-Respondents	All	Response Rate
**China**	1000	1778	2778	36%
**Denmark**	1000	3000	4000	25%
**France**	1000	1381	2381	42%
**Germany**	1250	2538	3788	33%
**Italy**	1250	644	1894	66%
**USA**	1000	1041	2041	49%

Supplementary Table 3

	Men												Women											
	**Cheeses**			**Cured meat**			**Meat**			**Fruits and Vegetable**			**Cheeses**			**Cured meat**			**Meat**			**Fruits and Vegetable**		
**Elderly**	mean	SD		mean	SD		mean	SD		mean	SD		mean	SD		mean	SD		mean	SD		mean	SD	
China	8.07	2.04	a	7.47	2.06	a	8.41	1.53	a	8.60	1.29	a	8.16	1.77	a	7.75	1.99	a	8.26	1.55	a	8.53	1.39	a
Denmark	5.54	3.01	c	4.90	3.02	b	6.45	2.73	d	6.48	2.75	c	5.68	2.78	c	4.91	2.83	bc	7.05	2.52	abc	7.45	2.48	abc
France	6.33	3.08	abc	4.50	2.43	ab	4.83	2.40	cd	7.17	1.84	abc	6.55	2.62	c	4.75	2.88	c	6.99	2.33	bc	7.11	2.43	c
Germany	6.52	2.62	b	6.65	2.73	a	7.06	2.65	bcd	7.04	2.41	bc	6.89	2.77	bc	6.33	3.12	b	6.84	3.21	c	7.69	2.52	bc
Italy	7.44	1.90	ab	7.58	1.88	a	7.88	1.84	ab	7.97	1.79	a	7.58	2.34	ab	7.53	2.35	a	7.91	2.35	ab	8.10	2.11	ab
The USA	7.17	2.82	ab	6.45	3.04	a	7.68	2.50	abc	7.64	2.53	ab	6.52	2.99	c	5.22	2.92	c	6.65	2.92	c	7.19	2.66	c
**Adults**																								
China	8.26	1.92	a	7.97	2.21	a	8.61	1.62	a	8.77	1.44	a	8.18	1.92	a	7.97	2.12	a	8.43	1.59	a	8.70	1.56	a
Denmark	6.55	2.66	c	6.23	2.87	c	7.13	2.52	d	7.32	2.45	b	5.94	3.05	c	5.33	3.09	d	7.02	2.77	c	7.60	2.49	c
France	7.04	2.43	bc	6.11	2.76	c	7.31	2.23	cd	7.55	2.10	b	6.90	2.34	b	5.52	3.06	d	6.93	2.69	c	7.62	2.11	c
Germany	7.27	2.46	b	6.91	2.73	b	7.25	2.70	cd	7.59	2.39	b	7.35	2.63	b	6.92	2.87	bc	7.65	2.63	b	8.13	2.19	b
Italy	7.38	1.86	b	7.43	1.84	ab	7.74	1.90	bc	7.59	1.89	b	7.38	2.14	b	7.41	2.16	ab	7.84	2.11	b	8.14	1.63	b
The USA	8.18	2.27	a	7.92	2.31	a	8.21	2.16	ab	8.37	1.08	a	7.36	2.81	b	6.66	3.16	c	7.51	2.80	bc	7.82	2.53	bc
**Young**																								
China	7.89	2.20	a	7.47	2.19		8.37	1.60	a	8.68	1.62	a	8.22	1.82	a	7.67	2.22	a	8.39	1.56	a	8.98	1.24	a
Denmark	6.64	2.85	b	6.51	2.80		7.29	2.51	b	7.49	2.56	b												
France	6.00	2.84	b	5.94	2.81		6.79	2.48	b	6.79	2.47	b	6.81	2.54	ab	5.14	3.37	b	7.00	2.69	ab	7.77	2.29	ab
Germany													6.91	2.68	b	5.91	3.09	b	6.67	3.10	b	8.00	2.06	ab
Italy	6.54	2.10	ab	7.15	1.62		7.62	1.77	ab	7.50	1.70	ab	7.15	2.18	ab	7.13	2.21	a	7.60	2.17	ab	7.92	1.51	b
The USA	7.31	2.64	ab	6.48	3.00		7.34	2.86	ab	7.48	2.57	ab	6.83	2.97	b	6.22	3.19	ab	6.92	3.00	b	7.53	2.74	b

Sustainable certification Interest Scale: 10= Extremely interested, 9= Very interested; 8= Quite interested, 7= Somewhat interested; 6= Slightly interested; 5= Neither interested nor uninterested, 4= Slightly uninterested; 3= Somewhat uninterested; 2= Very uninterested, 3= Somewhat uninterested; 1= Extremely uninterested. Different letters indicate statistical differences related to gender differences obtained using a least significant difference test ($P < 0.05$). P-values were adjusted using Bonferroni's method.

Supplementary Table 4

A)

	Men														
	Cheeses			Cured meat			Meat			Fruits			Vegetables		
**Elderly**	mean	SD		mean	SD		mean	SD		mean	SD		mean	SD	
China	3.45	1.12	a	3.32	1.11	a	3.59	1.06	a	4.02	0.84	a	4.04	0.77	a
Denmark	3.04	0.64	c	2.68	0.91	b	2.84	0.71	b	3.37	0.66	b	3.47	0.76	b
France	2.83	0.41	abc	2.67	0.52	ab	2.83	1.17	ab	3.50	0.84	ab	3.50	0.84	ab
Germany	3.15	0.73	bc	2.69	0.90	b	2.65	0.86	b	3.46	0.69	b	3.55	0.71	b
Italy	3.00	0.68	c	2.71	0.86	b	2.62	0.92	b	3.62	0.76	b	3.71	0.77	b
The USA	3.37	0.90	ab	2.95	0.99	ab	3.31	0.99	a	3.65	0.94	ab	3.76	0.90	ab
**Adults**															
China	3.67	0.98	a	3.47	1.00	a	3.66	0.91	a	4.05	0.76	a	4.04	0.78	a
Denmark	3.25	0.90	bc	3.02	1.05	b	3.05	1.06	b	3.57	0.86	b	3.67	0.91	b
France	3.35	0.89	b	3.10	1.11	b	3.14	1.06	b	3.63	0.89	b	3.66	0.87	b
Germany	3.38	0.87	b	3.07	1.15	b	3.06	1.09	b	3.56	0.88	b	3.63	0.82	b
Italy	3.15	0.82	c	2.96	0.90	b	2.99	0.93	b	3.60	0.81	b	3.68	0.77	b
The USA	3.87	1.08	a	3.55	1.13	a	3.71	1.06	a	3.98	0.98	a	3.97	0.92	a
**Young**															
China	3.47	1.14		3.28	1.11		3.86	0.92	a	3.93	0.88		3.96	0.91	
Denmark	3.55	1.14		3.41	1.15		3.44	1.20	ab	3.64	1.05		3.80	1.06	
France	3.29	1.17		3.03	1.27		3.65	1.20	ab	3.79	1.01		3.79	0.91	
Germany															
Italy	3.08	0.74		3.04	0.82		3.00	0.90	b	3.73	0.83		3.54	0.81	
The USA	3.63	1.06		3.13	1.26		3.53	1.05	ab	3.89	0.99		3.82	1.05	

5= Much more, 4= A little more, 3= Same as before 2= A little less, 1= Much less. Different letters indicate statistical differences related to gender differences obtained using a least significant difference test ($P < 0.05$). P-values were adjusted using Bonferroni's method.

B)

	Women														
	Cheeses			Cured meat			Meat			Fruits			Vegetables		
**Elderly**	mean	SD		mean	SD		mean	SD		mean	SD		mean	SD	
China	3.57	0.85	a	3.53	1.07	a	3.65	1.02	a	3.93	0.97	a	3.76	0.89	
Denmark	2.68	1.04	bc	2.27	1.16	bc	2.64	1.09	bcd	3.50	0.51	ab	3.86	0.77	
France	3.14	0.76	bc	2.49	0.94	bc	2.63	0.99	bc	3.41	0.87	b	3.47	0.83	
Germany	2.97	0.67	c	2.32	0.97	c	2.21	0.95	d	3.41	0.84	b	3.54	0.78	
Italy	2.98	0.71	c	2.54	0.83	bc	2.44	0.84	cd	3.62	0.83	b	3.68	0.85	
The USA	3.22	0.91	b	2.70	0.98	b	2.94	0.93	b	3.46	0.89	b	3.57	0.87	
**Adults**															
China	3.75	1.00	a	3.51	1.07	a	3.78	1.07	a	4.19	0.80	a	4.23	0.79	a
Denmark	2.97	0.74	d	2.68	0.92	c	2.68	0.97	d	3.48	0.89	d	3.65	0.87	b
France	3.18	0.83	bc	2.73	1.11	c	2.70	1.08	d	3.52	0.87	cd	3.58	0.87	b
Germany	3.32	1.07	b	2.86	1.18	bc	2.96	1.24	c	3.70	0.94	b	3.72	0.91	bc
Italy	3.06	0.87	cd	2.80	1.00	c	2.84	1.01	cd	3.68	0.83	bc	3.80	0.82	bc
The USA	3.59	1.03	a	3.07	1.23	b	3.42	1.12	b	3.79	0.97	b	3.80	0.93	c
**Young**															
China	3.53	0.92	ab	3.61	1.06	a	3.69	0.81	a	4.10	0.81		4.08	0.89	
Denmark															
France	3.12	0.76	abc	2.79	1.13	b	2.79	1.08	b	3.65	0.95		3.65	0.81	
Germany	2.99	1.15	c	2.71	1.23	b	2.67	1.21	b	3.83	0.99		3.83	0.92	
Italy	3.18	0.92	bc	3.00	1.17	b	2.87	1.12	b	3.75	0.89		3.79	0.85	
The USA	3.63	0.99	a	2.99	1.16	ab	3.64	1.07	a	3.78	0.92		3.90	1.02	

5= Much more, 4= A little more, 3= Same as before 2= A little less, 1= Much less. Different letters indicate statistical differences related to gender differences obtained using a least significant difference test ($P < 0.05$). P-values were adjusted using Bonferroni's method.

Supplementary Table 5

A)

	Men																				
	Fresh cheeses			Aged cheeses			Cured meat			Pork			Beef			Fruits			Vegetables		
**Elderly**	mean	SD		mean	SD		mean	SD		mean	SD		mean	SD		mean	SD		mean	SD	
China	4.24	1.72	ab	4.05	1.69	ab	3.62	1.90	ab	4.49	1.51	a	4.46	1.31	a	5.22	1.40	ab	5.46	1.75	a
Denmark	3.79	1.16	bc	3.56	1.25	bc	3.43	1.29	a	4.24	1.16	a	4.45	1.06	a	4.86	0.93	b	4.92	0.91	b
France	4.00	1.55	abc	4.00	1.67	abc	3.83	1.47	abc	3.67	1.63	abc	4.00	1.67	ab	5.17	0.41	abc	4.67	0.52	abc
Germany	4.39	1.21	a	4.07	1.33	ab	4.38	1.26	bc	3.60	1.50	b	3.60	1.42	b	4.96	1.05	b	4.96	1.01	ab
Italy	4.64	0.86	a	4.45	0.86	a	4.46	0.95	ab	4.31	1.06	a	4.53	0.92	a	5.50	0.85	a	5.42	0.94	ab
The USA	3.65	1.62	c	3.29	1.70	c	3.11	1.67	c	3.01	1.79	c	3.35	1.83	b	3.91	2.01	c	3.87	1.95	c
**Adult**																					
China	4.61	1.52	ab	4.42	1.49	a	4.21	1.82	c	5.03	1.25	a	4.88	1.23	a	5.28	1.30		5.43	1.38	a
Denmark	4.35	1.38	b	4.02	1.43	b	4.00	1.56	c	4.34	1.54	bc	4.60	1.39	ab	5.12	1.17		5.18	1.11	ab
France	4.60	1.26	ab	4.44	1.34	a	4.34	1.36	bc	4.06	1.60	c	4.50	1.27	b	5.18	1.13		5.21	1.09	ab
Germany	4.83	1.23	a	4.48	1.29	a	4.71	1.36	a	4.34	1.48	bc	4.42	1.46	b	5.11	1.03		5.12	1.04	b
Italy	4.77	0.88	a	4.47	0.91	a	4.59	0.92	ab	4.53	0.96	b	4.65	0.91	ab	5.24	0.96		5.32	0.87	ab
The USA	4.77	1.53	a	4.45	1.68	a	4.33	1.70	bc	4.25	1.83	bc	4.65	1.62	ab	5.06	1.62		5.06	1.62	b
**Young**																					
China	4.23	1.57		4.25	1.47		3.77	1.70		5.14	1.23	a	4.84	1.16		5.49	1.18		5.82	1.31	a
Denmark	4.65	1.45		4.54	1.47		4.45	1.53		4.62	1.53	bc	4.82	1.44		5.19	1.20		5.18	1.32	b
France	4.62	1.07		4.35	1.48		4.47	1.50		3.91	1.78	c	4.56	1.67		5.15	1.21		4.94	1.01	b
Germany																					
Italy	4.81	0.94		4.65	1.06		4.69	1.09		4.69	1.05	b	4.69	0.88		5.50	0.86		5.58	0.95	ab
The USA	4.42	1.77		4.00	2.00		3.87	1.95		4.11	2.00	b	4.48	1.77		5.06	1.66		4.89	1.66	b

B)

	Women																				
	Fresh cheeses			Aged cheeses			Cured meat			Pork			Beef			Fruits			Vegetables		
**Elderly**	mean	SD		mean	SD		mean	SD		mean	SD		mean	SD		mean	SD		mean	SD	
China	4.17	1.61	a	3.98	1.60	ab	3.44	1.78	b	4.57	1.43	a	4.31	1.47	a	5.00	1.45	ab	5.31	1.68	a
Denmark	3.68	1.39	ab	3.27	1.61	bc	3.36	1.33	ab	3.82	1.10	abc	4.50	1.14	a	5.14	0.56	ab	5.18	0.59	a
France	4.26	1.20	ab	4.03	1.13	ab	3.89	1.29	ab	3.52	1.38	bc	4.16	1.26	a	5.08	1.13	ab	5.16	1.07	a
Germany	4.36	1.11	b	3.78	1.35	b	4.10	1.35	a	3.19	1.54	c	3.05	1.31	b	5.06	0.90	b	5.03	0.87	a
Italy	4.73	0.97	bc	4.34	1.02	a	4.29	1.14	a	3.89	1.27	b	4.25	1.16	a	5.48	1.03	a	5.41	1.00	a
The USA	3.32	1.65	c	2.78	1.62	c	2.43	1.49	c	2.49	1.56	d	3.21	1.83	b	3.88	2.04	c	3.89	2.02	b
**Adult**																					
China	4.57	1.53	ab	4.41	1.53	a	4.08	1.77	b	4.99	1.25	a	4.86	1.18	a	5.47	1.16	a	5.72	1.22	a
Denmark	3.92	1.31	c	3.63	1.45	b	3.45	1.57	c	3.78	1.51	cd	4.19	1.38	c	5.17	1.00	b	5.40	0.89	b
France	4.43	1.19	b	4.19	1.29	a	3.93	1.41	b	3.63	1.60	d	4.09	1.38	c	5.12	1.06	b	5.13	1.04	c
Germany	4.74	1.31	a	4.29	1.60	a	4.53	1.49	a	3.96	1.77	bc	4.21	1.71	bc	5.46	1.05	a	5.41	1.06	b
Italy	4.76	1.04	a	4.46	1.11	a	4.51	1.17	a	4.23	1.28	b	4.48	1.18	b	5.48	1.12	a	5.54	1.05	ab
The USA	4.32	1.57	b	3.75	1.83	b	3.50	1.85	c	3.65	1.85	cd	4.16	1.68	bc	4.83	1.63	c	4.88	1.63	c
**Young**																					
China	4.49	1.35		4.55	1.42	a	4.06	1.59	ab	5.55	0.88	ab	4.73	1.10	a	5.76	0.99	a	5.98	0.86	a
Denmark																					
France	4.12	1.18		4.19	0.98	ab	4.14	1.30	ab	3.72	1.55	ab	4.07	1.06	ab	5.14	0.94	b	5.12	0.93	bc
Germany	4.50	1.61		4.03	1.59	ab	4.07	1.79	ab	3.28	2.04	b	3.72	1.70	b	5.35	0.98	ab	5.41	1.00	b
Italy	4.62	1.07		4.53	1.10	a	4.63	1.21	a	4.28	1.38	a	4.60	1.26	a	5.38	0.82	ab	5.31	0.91	b
The USA	4.56	1.40		3.75	1.84	b	3.74	1.78	b	4.15	1.72	ab	4.40	1.25	a	4.99	1.23	b	4.72	1.48	c

7= More than 5 times *per* week, 6= 3- 5 timer *per* week, 5= 1-3 times *per* week, 4= Once a month, 3= Once a year, 2= Special occasions, 1= Never. Different letters indicate statistical differences related to gender differences obtained using a least significant difference test ($P < 0.05$). P-values were adjusted using Bonferroni's method.
